# Supplementary material for: Are sites with multiple single nucleotide variants in cancer genomes a consequence of drivers, hypermutable sites or sequencing errors?
Source: PeerJ. 2016 Sep 20;4:e2391. doi: 10.7717/peerj.2391 (PMC5036107; doi:10.7717/peerj.2391)
Supplement: Table S1 — Excess SNVs from liver cancers split between the two labs of origin. RK indicates SNVs from the RIKEN lab and HX from the NCC. Significant heterogeneity of excess sites originating from different labs was tested using fishers exact test (see methods). [file peerj-04-2391-s001.doc]

**Supplementary table 1.**

Excess SNVs from liver cancers split between the two labs of origin. RK indicates SNVs from the RIKEN lab and HX from the NCC. Significant heterogeneity of excess sites originating from different labs was tested using fishers exact test (see methods).
